# Integrated metabolomics and transcriptomic analysis of the flavonoid regulatory networks in *Sorghum bicolor* seeds

**DOI:** 10.1186/s12864-022-08852-7

**Published:** 2022-08-26

**Authors:** Yaxing Zhou, Jingbo Lv, Zhonghao Yu, Zhenguo Wang, Yan Li, Mo Li, Zhilan Deng, Qingquan Xu, Fengjuan Cui, Wei Zhou

**Affiliations:** 1 Agricultural College, Inner Mongolia Minzu University, No. 996 Xilamulun Street, Kerqin District, Tongliao, 028000 Inner Mongolia People’s Republic of China; 2 Tongliao Agriculture and Animal Husbandry Research Institute, Tongliao, 028000 Inner Mongolia People’s Republic of China

**Keywords:** Flavonoid, Transcriptome, Metabolite, Sweet sorghum, Seed

## Abstract

**Background:**

The objective of this study was to reveal the flavonoid biosynthesis pathway in white (Z6), red (Z27) and black (HC4) seeds of the sweet sorghum (*Sorghum bicolor*) using metabolomics and transcriptomics, to identify different flavonoid metabolites, and to analyze the differentially expressed genes involved in flavonoid biosynthesis.

**Results:**

We analyzed the metabolomics and transcriptomics data of sweet sorghum seeds. Six hundred and fifty-one metabolites including 171 flavonoids were identified in three samples. Integrated analysis of transcriptomics and metabolomics showed that 8 chalcone synthase genes (*gene19114, gene19115, gene19116, gene19117, gene19118, gene19120, gene19122 and gene19123*) involved in flavonoid biosynthesis, were identified and play central role in change of color. Six flavanone including homoeriodictyol, naringin, prunin, naringenin, hesperetin and pinocembrin were main reason for the color difference.

**Conclusions:**

Our results provide valuable information on the flavonoid metabolites and the candidate genes involved in the flavonoid biosynthesis pathway in sweet sorghum seeds.

## Introduction

Sweet sorghum (*Sorghum bicolor*) is one of the most important cereal crops in the world and is remarkable for its resistant to stress and extremely high photosynthetic efficiency. Therefore, sweet sorghum could achieve high yields when it widely grown in harsh environments such as arid or semi-arid areas [1]. As a C4 drought resistant species, sweet sorghum has a huge potential for bioenergy [2], and is considered to be an ideal candidate for biofuel production due to its high efficiency of photosynthesis and the ability to maintain yield under harsh environmental conditions [3]. The harvest sweet sorghums used for industrial ethanol and densified biofuel production could combine soil remediation with creating economic benefit [4].

In our research, the seeds of three cultivars are selected. These three colors are the representative colors of all sorghum colors, that is, other sorghum colors are transition colors of these three. We know that the comprehensive agronomic traits of these three colors of sorghum are better. Previous studies found that the content and type of anthocyanin are major determinant of seed color, which is important natural colorants, and is widely found in leaves, flowers, fruits, and roots [5, 6]. The anthocyanins are beneficial to plants from UV radiation and pathogen, and are a natural antioxidants [7]. Furthermore, anthocyanin play an important role in antioxidant properties and enhanced nutritional value for plant seed [8]. Meanwhile, anthocyanins contributes to the seed pigmentation, and the high level of anthocyanin on top of phenolic content contributes to the greater pharmacological capacities [9]. Model species have been used to identify functional and regulatory genes involved in the flavonoid biosynthesis pathway in which two main classes of functional enzymes were identified. The “early biosynthetic step” enzymes can catalyze all flavonoid synthesis, and “later biosynthetic step” enzymes play important role in synthesis of flavonols and anthocyanins [10, 11].

In the current study, our aim was to identify significantly differentially expressed genes and metabolites involved in flavonoid biosynthesis pathway in the seeds of three sweet sorghum cultivars. Furthermore, we conducted research on the regulatory networks of flavonoid biosynthesis in seeds of sweet sorghum using metabolomics and transcriptomics. Our results not only provide candidate genes but also valuable information for metabolic engineering of flavonoid biosynthesis in seeds of sweet sorghum.

## Materials and methods

### Plant materials and sample preparation

The source of sweet sorghum cultivars (namely Z6, Z27, and HC4) from Agricultural College of Inner Mongolia Minzu University (N42°15’-45°41’, E119°15’-123°43’) in Tongliao City (Inner Mongolia, China) with excellent quality characteristics were chosen. Seeds of these three cultivars are white (Z6), red (Z27) and black (HC4), respectively. The experiments were conducted at the Experimental Base of the Agricultural College of Inner Mongolia Minzu University (N42°15’-45°41’, E119°15’-123°43’). The trial used a randomized block design with 3 replicates in planting 16 rows per plot. The sweet sorghum is bagged at the flowering stage, and mesh bag is alternative at the end of the flowering period. After maturity, the sweet sorghum were harvested. All sweet sorghum cultivars were sown on April 28, 2018 [12].

Seeds for each cultivar were collected from 5–6 plantlets as one sample. Each sample was split into two equal parts: one-half was used for metabolomic analysis and the other half for transcriptomic analysis. All the materials were frozen in liquid nitrogen immediately and then stored at -80 ℃ until use.

### Metabolite extraction

The frozen seeds were crushed using a bead beater (1.5 min, 30 Hz, three repetitions, MM 400, Retsch). One hundred milligrams of the powdered sample was extracted overnight at 4 ℃ using 1 mL of 70% aqueous methanol containing 0.1 mg·L^−1^ lidocaine. After centrifugation at 10 000 g for 10 min, the supernatants were filtrated by using 0.22 μm hydrophilic poly-(tetrafluoroethylene) syringe filters (SCAA-104, ANPEL, Shanghai, China) (http://www.anpel.com.cn/) before metabolomics analysis [13]. The quality control samples (mix1-3) were injected every three experimental samples throughout the analytical run to provide a set of data from which repeatability could be assessed.

### Metabolite profiling using liquid Chromatography-Electrospray Ionization-Tandem Mass Spectrometry (LC–ESI–MS/MS)

Metabolite profiling was conducted using a LC–ESI–MS/MS system (HPLC, UFLC SHIMADZU CBM30A system; MS, Applied Biosystems 4500 Q TRAP) (http://www.shimadzu.com.cn/) and an Agilent 6520 accurate-mass time-of-flight mass spectrometer (http://www.appliedbiosystems.com.cn/). Chromatographic separation was performed on an ACQUITY UPLC HSS T3 C18 column (2.1 mm × 100 mm × 1.8 μm; Waters) using mobile phase A (0.04% acetic acid in deionized water) and mobile phase B (0.04% acetic acid in acetonitrile). The elution profile was used as follows: 95:5 v(A)/v(B) at 0 min, 5:95 v(A)/v(B) at 11.0 min, 5:95 v(A)/v(B) at 12.0 min, 95:5 v(A)/v(B) at 12.1 min, and 95:5 v(A)/v(B) at 15.0 min. The flow rate was maintained at 0.4 mL/min. Mass data acquisition was performed in electrospray ionization positive/negative mode using the following parameters: ion spray voltage of ( ±) 5.5 kV; ion source gas I of 55 psi; gas II of 60 psi; curtain gas of 25 psi; turbo spray temperature of 550 ℃. Instrument tuning and mass calibration were performed with 10 and 100 μmol/L polypropylene glycol solutions in triple quadrupole and linear ion trap modes, respectively. Declustering potential (DP) and collision energy (CE) for individual multiple reaction monitoring (MRM) transitions were performed with specific DP and CE optimization. A specific set of MRM transitions were monitored for each period based on the metabolites eluted within this period [14, 15].

### Qualitative and quantitative analysis of metabolites

To facilitate the identification of metabolites by widely targeted metabolomics approach (MetWare, Wuhan, China), accurate m/z value of each precursor ions (Q1) were obtained [16]. This method has been previously described [14]. In brief, metabolites were identified by comparing the m/z values, the retention time (RT), and the fragmentation patterns with the standards in a self-compiled database (MetWare). Significantly changed metabolites (SCMs) were filtered according to |Log2 (fold change)|≥ 1, *p*-value < 0.05.

### RNA sequencing

RNA isolation, purification and monitoring, cDNA library construction and sequencing were performed as previously described [17]. Briefly, RNA purity, concentration and integrity were checked, measured and assessed using the NanoPhotometer® spectrophotometer (IMPLEN, Westlake Village, CA, USA), Qubit® RNA Assay Kit in Qubit® 2.0 Flurometer (Life Technologies, Carlsbad, CA, USA) and RNA Nano 6000 Assay Kit of the Agilent Bioanalyzer 2100 system (Agilent Technologies, Santa Clara, CA, USA), respectively [18]. Sequencing libraries were generated using NEBNext® Ultra™ RNA Library Prep Kit for Illumina® (NEB, Ipswich, MA, USA) following manufacturer’s recommendations and were sequenced on an Illumina Hiseq platform. According to the manufacturer’s instructions, NEBNext® Ultra™ RNA Library Prep Kit for Illumina® (NEB, Ipswich, MA, USA) was used to generate sequencing libraries, which were then sequenced on an Illumina Hiseq platform [18]. The clean data of RNA-seq were available from National Center for Biotechnology Information Sequence Read Archive database (SRA accession numbers: SRR8662425, SRR8662424).

### RT-qPCR (Real-Time Quantitative PCR)

The testing of RNA quality and determination of RNA concentration were performed by 1.0% agarose gel electrophoresis and micro ultraviolet spectrophotometry (Thermo NanoDrop 2000, Thermo Fisher Scientific, Waltham, MA, USA), respectively. Approximately 1 µg of total RNA was determined for cDNA synthesis using RevertAid™ First Strand cDNA synthesis kit (Thermo Fisher Scientific, Waltham, MA, USA). The LightCycler® 480 real-time PCR system with a 96-well plate was used to conduct an amplified reaction consisting of 95 ℃ for 5 min, followed by 45 cycles of 10 s at 95 ℃, 20 s at 60 ℃, and 20 s at 72 ℃ in a volume of 10 µL. At the end of each experiment, a melt-curve analysis was carried out using the default parameters (5 s at 95 ℃ and 1 min at 65 ℃). The β-actin was used for normalization [19, 20]. All analyses were repeated three times using biological replicates.

### Integrative analysis of metabolome and transcriptome

Metabolites and DEGs involved in phenylpropanoids biosynthesis and lipids metabolism in KEGG pathways were selected for integrative analysis. Metabolites used for correlation analysis were filtered according to variable importance in the project (VIP) > 1, *p*-value < 0.05, and |Log2 (Fold Change)|≥ 1. Pearson correlation coefficients and p-values were calculated for metabolome and transcriptome data integration using the spearman method [21].

### Statistical analysis

The relative expressions were calculated using the 2^−△△Ct^ method [22], and GraphPad Prism 5 (GraphPad Software Inc., San Diego, CA, USA) was used for chart preparation. The R (www.rproject.org/) and MEGA6 were used to conduct the heatmap and cluster analysis. Principle component analysis (PCA) was performed by R to study gene variety-specific accumulation. IBM SPSS Statistics 20 was used to test significant differences.

## Results

### Morphological differences among three sweet sorghum cultivars

Though the three sweet sorghum cultivars were planted simultaneously and grown in the same field and under the same conditions, there were obvious differences in the morphology of three cultivars, especially the color of the seeds.

**Fig. 1:**
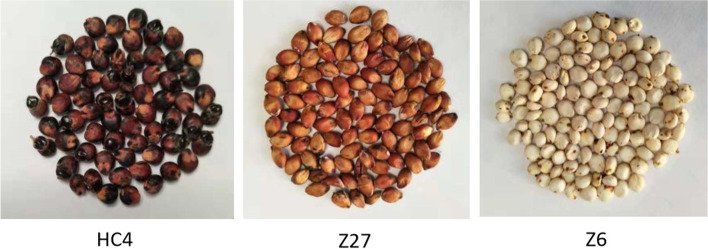
Seed color of the three sweet sorghum cultivars

Z6 was white, and Z27 and HC4 possess red/black (Fig. 1). Meanwhile, the research indicated that spike length, shank length and cutin rate in HC4 were higher than Z6 and Z27.

### Comprehensive analysis of metabolites

**Fig. 2:**
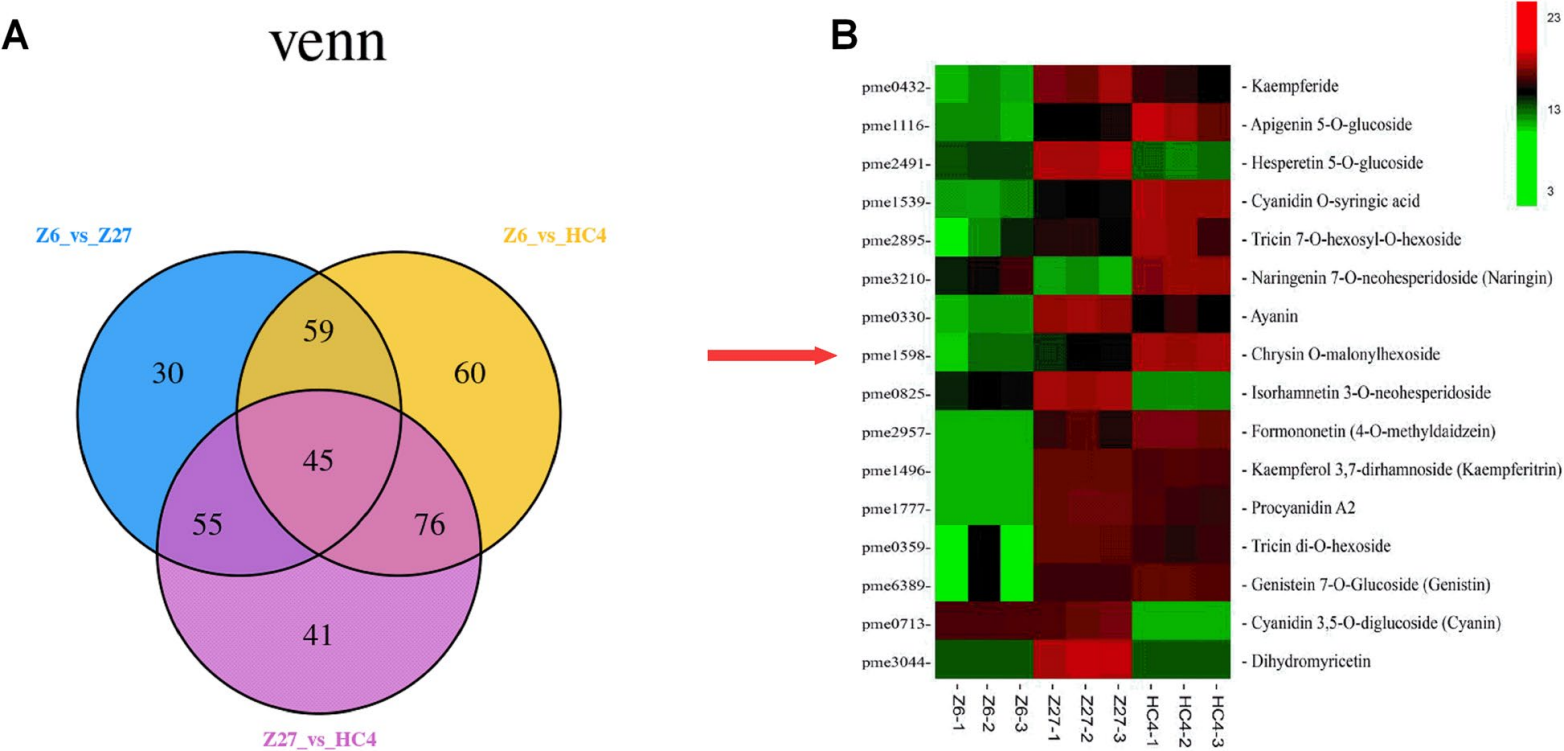
Venn Diagram result in three pairs of comparison groups. Differentially accumulated metabolites in three pairs of comparison groups

In order to explore the differences of metabolites in three sweet sorghum cultivars seeds (Fig. 1), a metabolome program was carried out. Six hundred and fifty-one metabolites were identified in three samples, including 34 flavonol, 58 flavone, 4 proanthocyanidins, 64 organic acids, 13 anthocyanins, and 29 amino acids*.* Then, according to the quantitative results of the identified metabolites, the differential metabolites between different group comparisons were analyzed based on fold-change and *p*-value. In the comparison between Z27 and HC4, a total of 111 and 106 metabolites presented as being up-regulated and down-regulated. Compared with HC4, Z6 had 182 metabolites up-regulated and 58 metabolites down-regulated. Compared with Z27, Z6 had 135 metabolites up-regulated and 54 metabolites down-regulated, respectively (Fig. 2A).

### Analysis of anthocyanins

Flavonoids, including anthocyanins, flavanes, flavones, flavanones, flavonols are found in numerous colored fruits and play an important role in pigmentation [23]. One hundred seventy-one flavonoids were found in three samples and many of flavonoids were differentially expressed in each comparison group. Seventy-five flavonoids were differentially expressed between Z6 vs. HC4. In HC4, procyanidin A1 and Procyanidin A2 were found with 24,740- and 5429- fold increments compared to Z6. Meanwhile, compared with Z6, 8 anthocyanins including, cyanidin 3,5-o-diglucoside (cyanin), cyanidin 3-o-glucosyl-malonylglucoside, cyanidin o-malonyl-malonylhexoside, cyanidin o-syringic acid, cyanidin o-acetylhexoside, cyanidin o-diacetyl-hexoside-o-glyceric acid, cyanidin 3-o-glucoside (kuromanin) and pelargonidin 3-o-beta-D-glucoside (callistephin chloride), significantly highly expressed in HC4.

Similarly, compared with Z6, procyanidin A3, procyanidin A2, procyanidin B2 and procyanidin B3 were increased 820,740-, 28,851-, 1958-, and 1,466,666- fold in the Z27 samples. Furthermore, 6 anthocyanins were identified and differentially expressed, of which peonidin O-hexoside, cyanidin o-syringic acid, delphinidin, cyanidin 3,5-o-diglucoside (cyanin) and pelargonidin 3-o-beta-d-glucoside (callistephin chloride) were up-regulated, and only delphinidin 3-o-glucoside (mirtillin) was down-regulated.

Ten anthocyanins were identified and differentially expressed in Z27 vs. HC4. Compared with Z27, peonidin, delphinidin and cyanidin 3,5-O-diglucoside (cyanin) were evidently down-regulated in HC4 group. Seven other anthocyanins were up-regulated in HC4.

### Flavonol, flavone, and flavanone

Fourteen flavones, 15 flavonols and 9 flavanones were differentially expressed in Z6 vs. Z27, among which syringetin 7-o-hexoside, dihydromyricetin, luteolin o-eudesmic acid-o-hexoside, myricetin showed 188,518-, 23,444-, 9088- and 8433-fold increases in Z27 with maximal difference. Twenty-one flavones, 12 flavonols and 10 flavanones were screened and differentially expressed in Z6 vs. HC4. Biochanin, myricetin and quercetin 5-o-malonylhexosyl-hexoside contents were 45,111-, 30,962- and 26,740-fold higher, respectively, in the HC4 vs. Z6.

In Z27 and HC4 comparison group, we identified 19 flavonol in which only myricetin, kumatakenin Rhamnetin (7-O-methxyl quercetin), and fustin were up-regulated in HC4 group. Twenty-nine flavone were found in HC4, most of which were up-regulated in HC4. Six flavanone were screened in HC4, in which eriodictyol and hesperetin 5-O-glucoside were up-regulated and others 4 flavanone were down-regulated.

Furthermore, we screened 16 DEMs from 45 common DEMs, including 1 procyanidine, 5 flavonol, 2 isoflavone, 3 flavanone, 4 flavone, and 1 anthocyanin, which were differentially expressed in three groups. The DEMs maybe play important roles in difference of color (Fig. 2B).

### Metabolic pathways: Flavonoid biosynthesis and flavone and flavonol biosynthesis

**Fig. 3:**
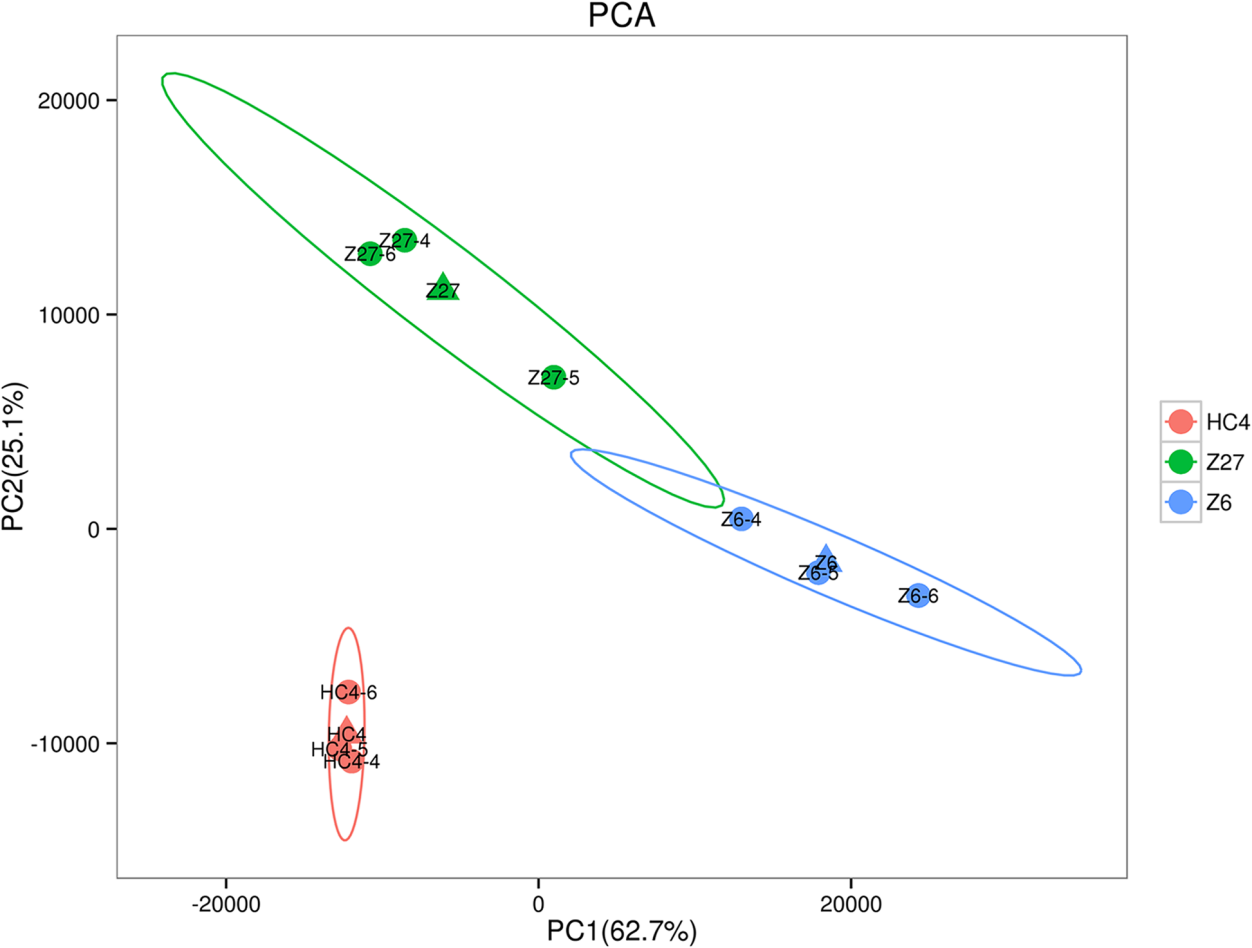
PCA scores plot of different cultivars. PC1, principal component 1; PC2, principal component 2. Explained variants PC1: 62.7%, PC2: 25.1%

**Fig. 4:**
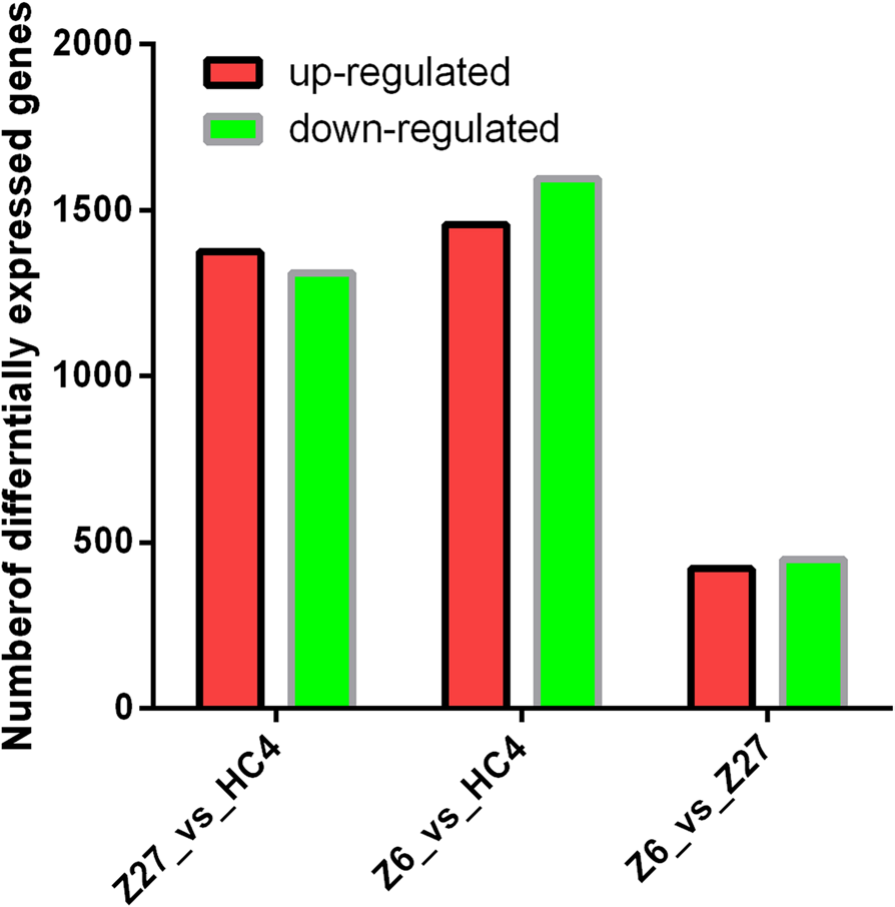
Numbers of differentially expressed genes

Principal component analysis (PCA) was performed indicating that the score plots of PCA (Fig. 3) exhibited an obvious separation in 3 cultivars. The two principal components (PC1 and PC2) were 62.7% and 25.1%, respectively. The PCA clusters indicated that the three cultivars were distinct. Then, we compared the differentially expressed genes (DEGs) in every two cultivars. The number of differentially expressed genes had very high variance among different cultivars. In total, 3054 DEGs were identified including 1458 up-regulated and 1596 down-regulated genes, which were differentially expressed at Z6 vs. HC4. There were 1375 up-regulated DEGs and 1312 down-regulated DEGs at Z27 vs. HC4, 422 up-regulated DEGs and 450 down-regulated DEGs in Z6 vs. Z2 (Fig. 4).

KEGG analysis revealed flavonoid biosynthesis and flavone and flavonol biosynthesis as the significantly changed pathways. Furthermore, the related DEGs in the pathway were screened in Z6 vs. Z27, Z27 vs. HC4 and Z6 vs. HC4 (Table 1).

**Table 1:** KEGG enrichment pathways in different comparison groups

Comparison group	pathway	ko_ID	DEG	gene ID	up/down
Z6_vs_Z27	Flavone and flavonol biosynthesis	ko00944	1	*gene16076*	Down
Z6_vs_Z27	Flavonoid biosynthesis	ko00941	3	*gene16076;gene24313;gene344*	Down
Z27_vs_HC4	Flavone and flavonol biosynthesis	ko00944	4	*Sorghum_bicolor_newGene_10649;gene16073;gene16076;gene16077*	Up
Z27_vs_HC4	Flavonoid biosynthesis	ko00941	21	*Sorghum_bicolor_newGene_10137;Sorghum_bicolor_newGene_10649;gene14509;gene15470;gene16073;gene16076;gene16077;gene17170;gene19114;gene19115;gene19116;gene19117;gene19118;gene19120;gene19122;gene19123;gene26712;gene30278;gene344;gene6640;gene7823*	Up
Z6_vs_HC4	Flavone and flavonol biosynthesis	ko00944	1	*Sorghum_bicolor_newGene_10649*	Up
Z6_vs_HC4	Flavonoid biosynthesis	ko00941	14	*Sorghum_bicolor_newGene_10137;Sorghum_bicolor_newGene_10649;gene15470;gene17170;gene19114;gene19115;gene19116;gene19117;gene19118;gene19120;gene19122;gene19123;gene26712;gene30278*	Up
Z6_vs_HC4	Flavonoid biosynthesis	ko00941	1	*gene24313*	Down

### Correlation network graph analysis genes and metabolites

**Fig. 5:**
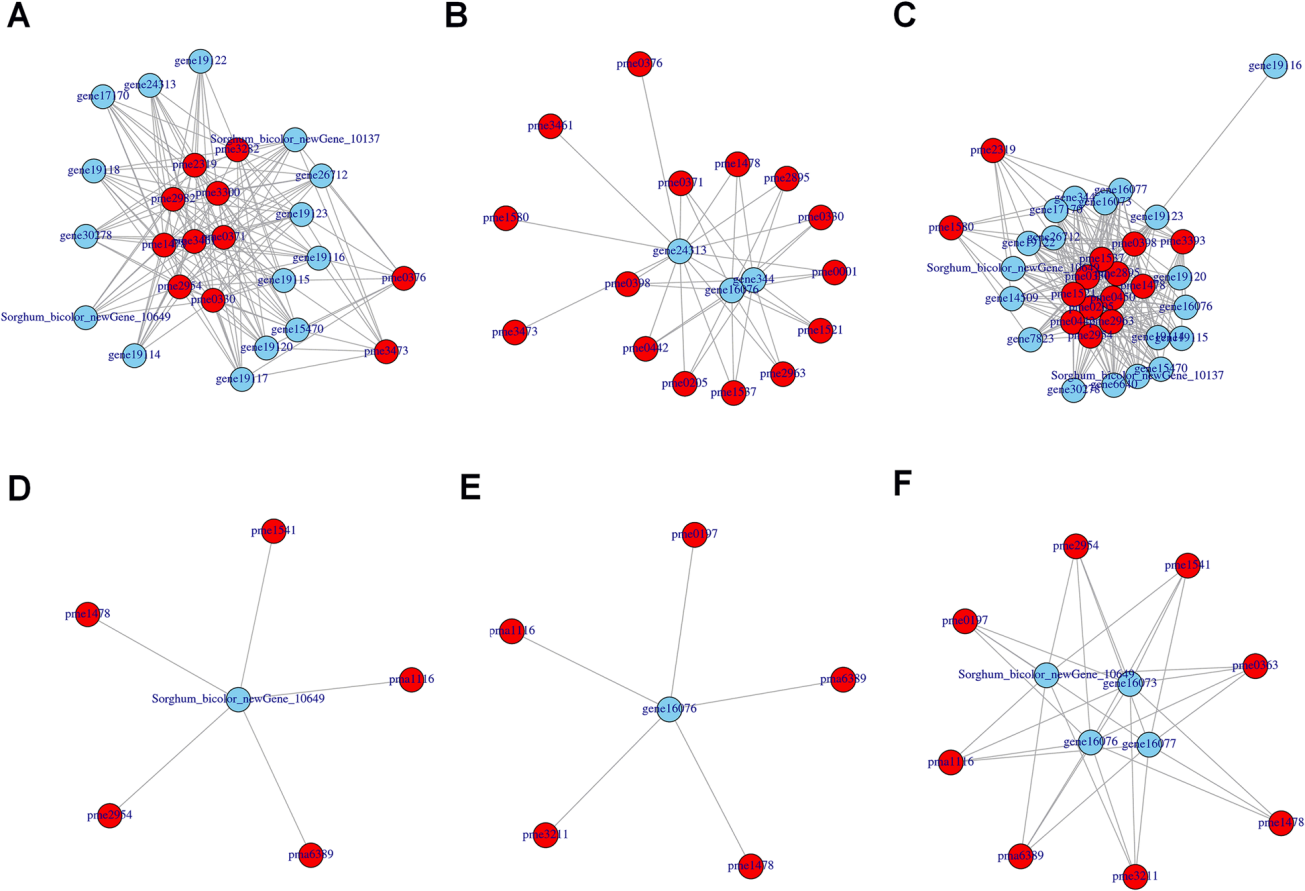
Gene and metabolite association analysis. In the ko00941 pathway, Gene and metabolite association analysis in Z6 vs. HC4 (**A**), Z6 vs.Z27 (**B**), and Z27 vs. HC4 (**C**). In ko00944 pathway, Gene and metabolite association analysis in Z6 vs. HC4 (**D**), Z6 vs. Z27 (**E**) and Z6 vs.HC4 (**F**)

Subsequently, the DEGs and DEMs in flavonoid biosynthesis and flavone and flavonol biosynthesis were screened. Pearson correlation coefficient analysis was performed on the genes and metabolites to explore regulatory mechanisms. In the ko00941 pathway, 15 genes were related to 11 metabolites between Z6 and HC4 (Fig. 5A). Accordingly, gene 24313, gene16076 and gene344 were screened, which was relevant to 15 metabolites between Z6 and Z27 (Fig. 5B). 14 metabolites were related to 18 genes in Z27 vs. HC4 (Fig. 5C).

In ko00944 pathway, Sorghum_bicolor_newGene_10649 was found, and it was relevant to 5 metabolites including pme1541, pma6389, pme1478, pme2954 and pma1116 in Z6 vs. HC4 (Fig. 5D). The gene16076 was negative correlation with 5 metabolites in Z6 vs. Z27 (Fig. 5E). Fifteen genes were related to 11 metabolites between Z6 and HC4 (Fig. 5F).

### Regulatory network of flavonoid biosynthesis

**Fig. 6:**
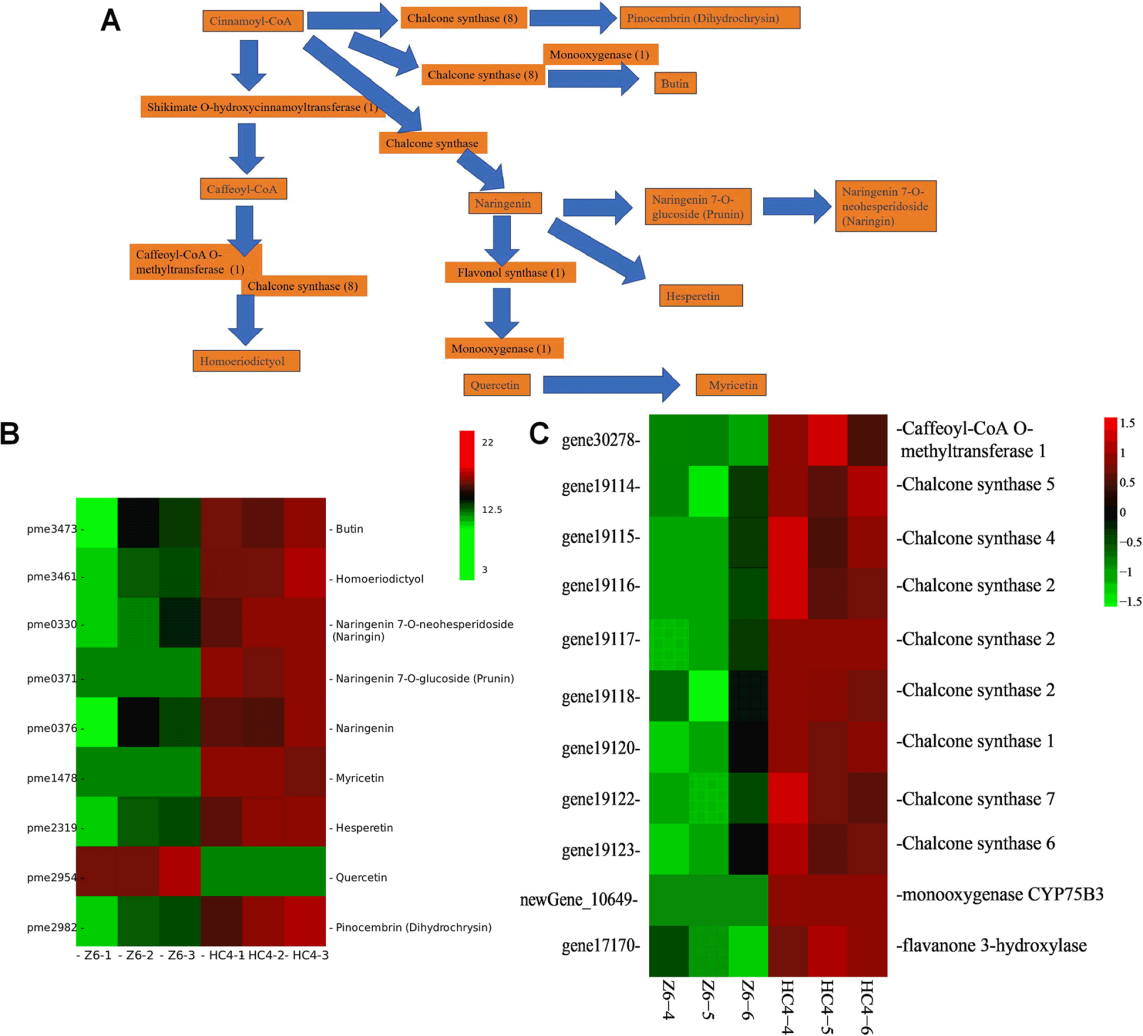
Key genes and metabolites involved in flavonoids biosynthesis pathway. **A** The flavonoids biosynthesis pathway. **B** The heatmap of key genes and metabolites

The color difference was greatest between Z6 and HC4, so the pathway of flavonoid biosynthesis were analyzed in two groups (Fig. 6A). Eight chalcone synthase genes (gene19114, gene19115, gene19116, gene19117, gene19118, gene19120, gene19122 and gene19123) involved in flavonoid biosynthesis, were identified and up-regulated in HC4 (Fig. 6B). Caffeoyl-CoA O-methyltransferase 1 and flavanone 3-hydroxylase showed twofold and 2.7-fold upregulation in the HC4 group.

Six flavanone including homoeriodictyol, naringin, prunin, naringenin, hesperetin and pinocembrin were high fold up-regulation in HC4 (Fig. 6C). Myricetin was one of the most significantly DEMs in the HC4 vs. Z6 group, increasing 30,962-fold. 1 flavone (butin) was upregulated 1.4-fold in HC4 vs. Z6.

### RT-qPCR validation of the transcriptomic data

**Fig. 7:**
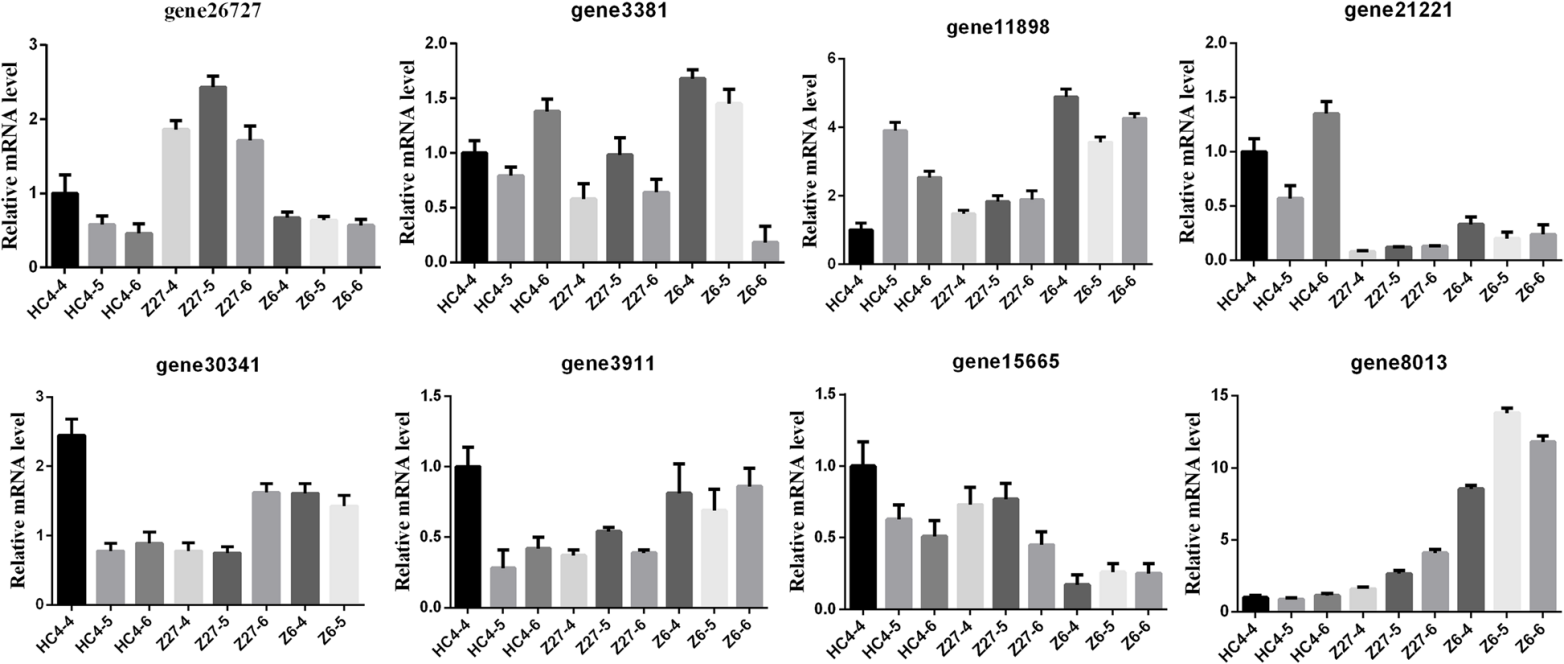
The expression of representative genes in 3 samples validated by qRT-PCR

To validate the key RNA-Seq results, we selected 8 genes and analyzed their expression levels in Z6, HC4 and Z27 using RT-qPCR. The results validated the relevance of the RNA-Seq data and RT-qPCR showed good consistency for both up- and down- regulated gene expression (Fig. 7).

## Discussion

The color of the sweet sorghum seed of different cultivars varies widely, mainly due to the fact that the seed coat contains different flavonoids. A combined metabolome and transcriptome study can provide us with new, large-scale information on the shifted flavonoids and the underlying modifications in gene-expression networks.

In our research, integrated metabolomics and transcriptomic analysis were employed to explore gene expression and flavonoid alterations for the color of the sweet sorghum seed of different cultivars. The results indicated that there were significant differences in the seed color of three cultivars, grown in the same conditions. Meanwhile, 651 metabolic components were identified including 34 flavonol, 58 flavone, 4 proanthocyanidins and 13 anthocyanins, which are commonly identified in pink, red, purple and other deep-colored fruit [24]. To understand the metabolic characteristics of the color of the sweet sorghum seed, our attention was focused on main flavonoid compounds in all samples.

Out results indicated that 171 flavonoids were found in three samples. 75 flavonoids were differentially expressed between Z6 vs. HC4. The procyanidin A1 and procyanidin A2 were evidently higher in HC4 compared to Z6. Eight anthocyanins including were significantly highly expressed in HC4. Meanwhile, the contents of procyanidin A3, procyanidin A2, procyanidin B2 and procyanidin B3 were higher in the Z27 samples. Flavonoids are composed of anthocyanins, flavonoids, chalcone, flavonoids, flavonols and isoflavones. Among them, anthocyanins are the main determinant of color formation in flower and fruit [25]. Previous study found that the cyanidin-3-O-rhamnoglucoside (cyanidin-3-O-rutinoside) was the main anthocyanin in the peel of “Black Mission,” “Bursa” and “Brown Turkey” figs [26, 27]. Acyl-modified anthocyanins have been identified in Arabidopsis [28]. Study indicated that the cyanidin 3-O-(malonyl)-glucoside was the only pigment responding to temperature in the cool-cultivated red lettuce [29]. Anthocyanins and flavonoids affect fruit color and taste [30]. We speculated that the darker color of seeds in Z27 and HC4 were mainly due to the higher anthocyanins content of these two groups.

In the present study, several critical genes were highly expressed in HC4, which play important roles in the pathway of flavonoid biosynthesis. Eight chalcone synthase genes (CHS) involved in flavonoid biosynthesis were identified and up-regulated in HC4. Chalcone synthase, a key enzyme in the flavonoid biosynthetic pathway, has been studied in many plants [31]. The structural gene expression levels of chalconesynthase (CHS) and chalcone isomerase (CHI) were highly similar and significantly positively correlated with anthocyanin accumulation rate in wild Lycium ruthenicum Murr [32]. Consistent with our study, in crabapple cultivars with dark red, pink and white petal colors, CHS play an important role in the formation of the red coloration [33]. In the white-flowered individuals, the expression of CHS gene was significantly inhibited, while the expression of other genes in the anthocyanin biosynthesis pathway was similar to that in pigmented individuals [34].

In summary, our study provide us insight on modulated anthocyanin and flavonoid expression in three sweet sorghum cultivars, revealing the large-scale changes in nutritionally important compounds and gene expression in seeds of three cultivars. Our results provide new information on the anthocyanidin, flavonol and procyanidin metabolites of sweet sorghum cultivars and the global transcriptional changes in seeds color regulation.

## Data Availability

The datasets generated and/or analyzed during the current study are available in National Center for Biotechnology Information Sequence Read Archive database (https://www.ncbi.nlm.nih.gov/sra/?term, SRA accession numbers: SRR8662425, SRR8662424).
